# Impact of Estimated Glomerular Filtration Rate and Serum C‐Reactive Protein Level to Overall Survival After Second‐Line Targeted Therapy Following Immuno‐Oncology Combination Therapy for Advanced Renal Cell Carcinoma

**DOI:** 10.1111/iju.70138

**Published:** 2025-05-28

**Authors:** Keita Nakane, Hiromitsu Watanabe, Taku Naiki, Kiyoshi Takahara, Teruo Inamoto, Takahiro Yasui, Ryoichi Shiroki, Hideaki Miyake, Takuya Koie

**Affiliations:** ^1^ Department of Urology Gifu University Graduate School of Medicine Gifu Gifu Japan; ^2^ Department of Urology Hamamatsu University School of Medicine Hamamatsu Shizuoka Japan; ^3^ Department of Nephro‐Urology, Graduate School of Medical Sciences Nagoya City University Nagoya Aichi Japan; ^4^ Department of Urology Fujita Health University School of Medicine Toyoake Aichi Japan; ^5^ Department of Urology Kobe University Graduate School of Medicine Kobe Hyogo Japan

**Keywords:** axitinib, cabozantinib, immune checkpoint inhibitor, renal cell carcinoma, second‐line therapy

## Abstract

**Objectives:**

Immune checkpoint inhibitor (ICI)‐based combination therapies are first‐line treatments for locally advanced or metastatic renal cell carcinoma (mRCC). However, second‐line treatment efficacy remains uncertain due to limited large randomized trials. This study evaluated real‐world oncological outcomes after second‐line treatments in patients who received combination ICIs as first‐line treatment.

**Methods:**

Among 467 patients who received ICI combination therapy as first‐line treatment for mRCC between January 2018 and January 2024, those who received cabozantinib (Cabo) or axitinib (Axi) as second‐line treatment were included in this study. The patient characteristics at the initiation of second‐line treatment, progression‐free survival (PFS), and overall survival (OS) were compared between the two groups. Prognostic factors associated with OS after the initiation of second‐line treatment were evaluated.

**Results:**

The Cabo and Axi groups included 87 and 45 patients, respectively. Median OS and PFS after the initiation of secondary treatment were 32 and 9 months in the Cabo group (*p* = 0.269), and 33 and 12 months in the Axi group (*p* = 0.399). Multivariable analysis identified serum C‐reactive protein (CRP) ≥ 0.6 mg/dL and estimated glomerular filtration rate (eGFR) < 40 mL/min/1.73 m^2^ at the start of secondary treatment as independent predictors of OS. Stratification by these factors revealed a significant OS difference (*p* < 0.001).

**Conclusions:**

Oncological outcomes after the initiation of secondary treatment did not differ significantly between the Cabo and Axi groups. An eGFR < 40 mL/min/1.73 m^2^ and CRP ≥ 0.6 mg/dL at the start of Cabo or Axi treatment were independent OS predictors after secondary treatment.

## 1 Introduction

While treatment with interferon and interleukin‐2 followed by molecularly targeted therapies alone has been used as first‐line treatment for locally advanced or metastatic advanced renal cell carcinoma (mRCC), combination therapy with immune checkpoint inhibitors (ICIs) is currently widely used [1, 2, 3, 4, 5, 6, 7]. The prognosis of patients with mRCC has improved significantly with the introduction of ICI combination therapy as first‐line treatment [1, 2, 3, 4, 5, 6, 7]. Although there are a growing number of reports on the therapeutic efficacy of subsequent second‐line regimens, the lack of randomized trials has resulted in a lack of consensus on the optimal sequential regimens [8, 9, 10, 11, 12, 13, 14, 15]. Therefore, this study included patients who received a vascular endothelial growth factor receptor‐tyrosine kinase inhibitor (VEGFR‐TKI) treatment as second‐line treatment after ICI combination therapy. Overall survival (OS) and progression‐free survival (PFS) from the initiation of the second‐line treatment were assessed using the Tokai Urological Oncology Seminar (TOURS) cohort. Additionally, clinical factors predictive of prolonged OS after the initiation of second‐line treatment were investigated.

## 2 Methods

### 2.1 Ethics

The trial was approved by the Medical Review Committee of the Gifu University Graduate School of Medicine (approval number: 2024‐043). Due to the retrospective nature of this study, informed consent was not obtained from all eligible patients and was replaced by an opt‐out option. According to the Japanese Ethics Committee and ethical guidelines, retrospective and cohort studies using existing literature and other sources do not require written consent because the research information is publicly available. More information on this retrospective cohort study, albeit in Japanese, is available at https://rinri.med.gifu‐u.ac.jp/esct/publish_document.aspx?ID=3093 (accessed November 11, 2024).

### 2.2 Patients

This retrospective, multicenter cohort study included 467 patients with mRCC who received ICI combination therapy as a first‐line treatment in a cohort of four academic centers that comprise the Tokai Urology Oncology Seminar (TOURS) and its affiliate institutions between January 2018 and January 2024. Since many patients received the VEGFR‐TKIs cabozantinib (Cabo) or axitinib (Axi) as sequential therapy, those who received these two agents as second‐line therapy after ICI combination therapy were included in this analysis. Patients who received other VEGFR‐TKIs and those with missing data were excluded. Age, sex, body mass index (BMI), Karnofsky performance status (KPS) [16], predominant tumor histology, International Metastatic RCC Database Consortium (IMDC) risk score at the initiation of first‐line treatment, metastatic sites [17], C‐reactive protein (CRP), serum creatinine, albumin (Alb) and calcium (Ca), estimated glomerular filtration rate (eGFR), enrolled patients receiving first‐line regimens, best overall response (BOR) at the first‐ and second‐line treatment, and duration of follow‐up were extracted from the medical records. Patients receiving Cabo or Axi as second‐line treatment were assigned to the Cabo or Axi arm, respectively. Patient background, OS, and PFS after the initiation of second‐line treatment were compared between the two groups. Additionally, the prognostic factors predictive of OS after the initiation of second‐line treatment were investigated using multivariable analysis.

### 2.3 Patient Evaluation

Computed tomography (CT) was used to assess tumor status before and after treatment. Imaging assessments were performed before and every 2–3 months after the initiation of combination ICI or VEGFR‐TKI therapy according to the policy of each institution and the treating physician. BOR after second‐line treatment was assessed using the Response Evaluation Criteria in Solid Tumors, Version 1.1, and was diagnosed as complete response (CR), partial response (PR), stable disease (SD), or a progressive disease (PD) [18]. The eGFR was calculated using the formula eGFR = 194 × serum creatinine^−1.094^ × age^−0287^ (for females, multiplied by 0.739) [19].

### 2.4 Statistical Analysis

All statistical analyses were performed using EZR version 1.56 (Saitama Medical Center, Jichi Medical University, Saitama, Japan) with a graphical user interface of R version 3.3.0 (The R Foundation for Statistical Computing, Vienna, Austria) [20]. OS was defined as the time from the initiation of second‐line treatments to death from RCC or any cause, and PFS was defined as the time from the initiation of second‐line treatments to disease progression or death. Continuous variables were analyzed using the Mann–Whitney *U* test, and categorical variables were analyzed using Pearson's chi‐squared test or Fisher's exact test. Oncological outcomes, including OS and PFS, were evaluated using the Kaplan–Meier method, and differences in clinical variables were evaluated using the log‐rank test. Multivariable analysis using the Cox proportional hazards model was performed to explore the prognostic factors for overall survival after the initiation of second‐line therapy. The optimal cutoff values for the explanatory variables were determined using the Youden index obtained from the receiver operating characteristic (ROC) curve. The number of explanatory variables included in the multivariable analysis was determined by dividing the total number of events by 10 [21]. A two‐tailed *p*‐value < 0.05 was considered statistically significant for all analyses.

## 3 Results

### 3.1 Patient Characteristics

**FIGURE 1:**
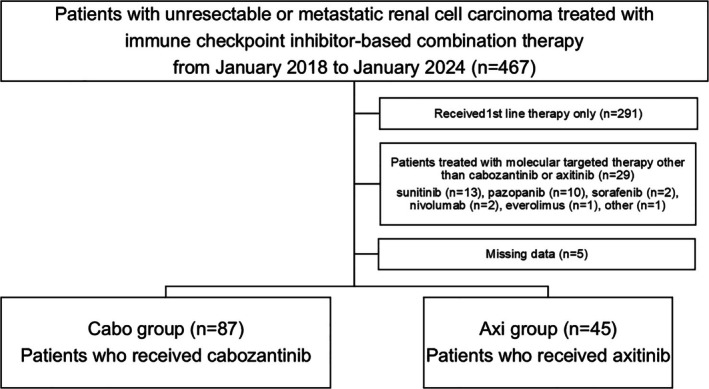
Flow diagram for patient selection.

During the inclusion period, a total of 467 patients were enrolled. Of the 467 patients, 291 who received only first‐line therapy, 29 who received second‐line therapy other than Cabo or Axi, and 5 with missing data were excluded. Finally, 87 and 45 patients were assigned to the Cabo and Axi groups, respectively (Figure 1). Table 1 shows the characteristics of the patients enrolled in this study at the time of first‐line therapy initiation. The median age of all patients enrolled was 69 years (interquartile range [IQR], 59–74 years) and BMI was 22.5 (IQR, 20.5–25.5). Among all enrolled patients, 21 patients (15.9%) had a KPS of ≤ 70%, and the most common histological type was clear cell carcinoma (79.5%). The median CRP, Alb, Ca, and eGFR of all enrolled patients were 0.60 mg/dL (IQR, 0.2–3.4), 3.7 g/dL (IQR, 3.2–4.0), 9.5 mg/dL (9.2–10.2), and 52.2 mL/min/1.73 m^2^ (IQR, 39.0–71.4), respectively. As the first‐line regimen, ipilimumab + nivolumab was administered to 68 patients (51.5%), while pembrolizumab + lenvatinib was administered to 12 (9.1%), nivolumab + Cabo to 11 (8.3%), and avelumab + Axi to 6 patients (4.5%), and the number of patients receiving these regimens was relatively small. The reason for the significant difference in the choice of first‐line treatment between the two groups was that patients who received first‐line treatment with Cabo or Axi tended to choose another VEGFR‐TKI as second‐line treatment. Among patients who received nivolumab + ipilimumab as first‐line therapy, we compared the backgrounds of patients who received cabozantinib as second‐line therapy and those who received axitinib as second‐line therapy. We found only a significant difference in the duration of the observation period (Table S1).

**TABLE 1:** Patient background.

Characteristics	Cabozantinib	Axitinib	*p*
Number	87	45	
Age at initiation of second‐line therapy (median, year, IQR)	68.0 (60.5–74.0)	73.0 (62.0–76.0)	0.233
Sex (number, %)			0.386
Male	65 (74.7)	37 (82.2)	
Female	22 (25.3)	8 (17.8)	
BMI at initiation of first‐line therapy (median, kg/m^2^, IQR)	22.6 (20.4–25.9)	22.2 (20.5–24.5)	0.411
KPS at initiation of first‐line therapy (number, %)			0.707
100	39 (45.3)	17 (37.8)	
90	22 (25.6)	12 (26.7)	
80	11 (12.8)	8 (17.8)	
70	14 (16.0)	7 (15.5)	
Unknown	0 (0.0)	1 (2.2)	
Predominant histology (number, %)			0.970
Clear cell	69 (82.1)	36 (90.0)	
Papillary	4 (4.5)	0 (0.0)	
Chromophobe	1 (1.2)	1 (2.5)	
Other	11 (12.8)	3 (6.6)	
Unknown	2 (2.2)	5 (11.1)	
IMDC risk at initiation of first‐line therapy (number, %)			0.240
Favorable	11 (12.8)	2 (4.4)	
Intermediate	46 (51.1)	24 (53.3)	
Poor	30 (33.3)	19 (42.2)	
Metastatic sites at initiation of first‐line therapy (number, %)			
Lung	55 (63.2)	34 (75.6)	0.174
Bone	23 (26.4)	15 (33.3)	0.424
Liver	12 (13.8)	5 (11.1)	0.665
Lymph node	12 (13.8)	15 (33.3)	0.012
Soft tissue	0 (0.0)	6 (13.3)	0.001
Adrenal gland	3 (3.4)	7 (15.6)	0.031
Brain	4 (4.5)	0 (0.0)	0.300
Pleura	1 (1.1)	2 (4.4)	0.268
Skeletal muscle	0 (0.0)	3 (6.6)	0.038
Local recurrence	0 (0.0)	3 (6.6)	0.038
Pancreas	1 (1.1)	0 (0.0)	1.000
Peritoneal carcinomatosis	2 (3.3)	1 (2.2)	1.000
C‐reactive protein (median, mg/dL, IQR)	0.60 (0.1–3.2)	0.80 (0.3–3.5)	0.371
Serum Albumin (median, g/dL, IQR)	3.7 (3.1–4.1)	3.6 (3.3–4.0)	0.790
Serum Calcium (median, mg/dL, IQR)	9.5 (9.2–10.0)	9.6 (9.4–10.5)	0.106
eGFR (median, ml/min/1.73 m^2^, IQR)	51.1 (38.9–76.3)	52.7 (40.6–70.3)	0.985
Previous surgical removal of primary site (number, %)			0.853
Yes	51 (58.6)	25 (55.6)	
No	36 (40.0)	20 (44.4)	
First‐line therapy (number, %)			< 0.001
Ipilimumab + Nivolumab	35 (40.2)	33 (73.3)	
Pembrolizumab + axitinib	34 (39.1)	1 (2.2)	
Pembrolizumab + lenvatinib	11 (12.6)	1 (2.2)	
Nivolumab + cabozantinib	1 (1.1)	10 (22.2)	
Avelumab + axitinib	6 (6.7)	0 (0.0)	
Reason for discontinuing first line‐therapy			
Disease progression	55 (64.0)	26 (57.7)	0.619
Adverse event	30 (34.9)	18 (40.0)	
Other	2 (2.2)	1 (2.2)	
Follow up period (months, IQR)	20.0 (13.0–28.0)	21.0 (13.0–37.0)	0.243

Abbreviations: BMI, body mass index; eGFR, estimated glomerular filtration rate; IMDC, international metastatic RCC database consortium; IQR, interquartile range; KPS, Karnofsky performance status.

### 3.2 Oncological Outcomes

The BOR for the first‐line treatment was 4.5% for CR, 47.0% for PR, 32.6% for SD, and 15.9% for PD (data not shown).

**FIGURE 2:**
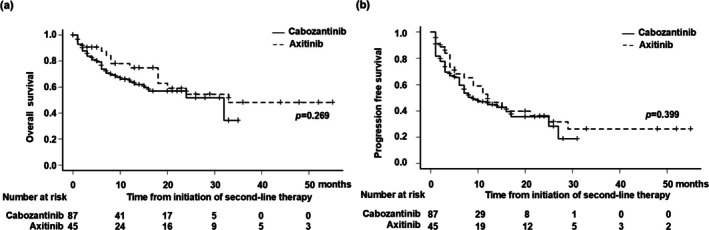
(a) Kaplan–Meier curve analysis of overall survival (OS) after initiation of second‐line therapy by vascular endothelial growth factor receptor‐tyrosine kinase inhibitor (VEGFR‐TKI) agent. The median OS was 32 months in patients receiving cabozantinib and 33 months in those receiving axitinib (*p* = 0.269). (b) Kaplan–Meier curve analysis of progression‐free survival (PFS) after initiation of second‐line therapy by VEGFR‐TKI agent. The median PFS was 8 months in patients receiving cabozantinib and 12 months in those receiving axitinib (*p* = 0.399).

At a median follow‐up of 21.0 months, 46 patients (34.8%) died from any cause. The median OS from the initiation of second‐line VEGFR‐TKI treatment was 32 months (95% confidence interval [95% CI], 13 months–not applicable [NA]) in the Cabo arm and 33 months (95% CI, 18 months–NA) in the Axi arm (Figure 2a). The 1‐ and 2‐year OS for the Cabo and Axi groups were 64.1% (95% CI, 51.7%–74.2%), 74.9% (95% CI, 56.8%–86.2%), 51.9% (95% CI, 36.0%–65.5%), and 54.6% (95% CI, 34.5%–70.8%), respectively (*p* = 0.269; Figure 2a). The median PFS from the start of the second‐line treatment with VEGFR‐TKIs was 9 months (95% CI, 6–17 months) in the Cabo arm and 12 months (95% CI, 7–25 months) in the Axi arm (*p* = 0.399; Figure 2b). The 1‐ and 2‐year PFS were 45.0% (95% CI, 33.0%–56.2%) and 35.6% (95% CI, 22.5%–48.9%) for the Cabo group, and 46.4% (95% CI, 29.5%–61.7%) and 36.1% (95% CI, 20.3%–52.2%) for the Axi group (Figure 2b). The oncological outcomes after the initiation of second‐line treatment were not significantly different between the Cabo and Axi groups.

**FIGURE 3:**
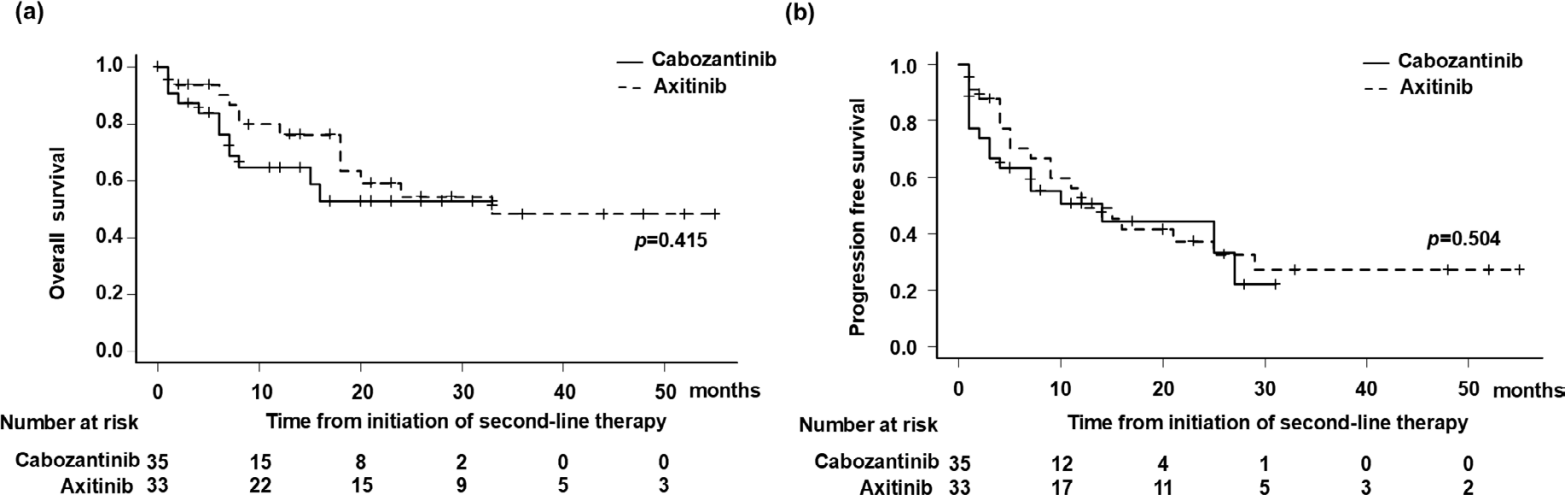
(a) Kaplan–Meier curve analysis of overall survival (OS) after initiation of second‐line treatment by vascular endothelial growth factor receptor‐tyrosine kinase inhibitor (VEGFR‐TKI) agent in patients whose first‐line treatment was nivolumab plus ipilimumab. Median OS was not reached in patients receiving cabozantinib and 33 months in those receiving axitinib (*p* = 0.415). (b) Kaplan–Meier curve analysis of progression‐free survival (PFS) after initiation of second‐line treatment by VEGFR‐TKI agent in patients whose first‐line treatment was nivolumab plus ipilimumab. Median PFS was 14 months in the patients receiving cabozantinib and 12 months in those receiving axitinib (*p* = 0.504).

To evaluate the impact of different first‐line regimens on second‐line treatment, oncological outcomes were compared between the Carbo and Axi groups of patients who received nivolumab plus ipilimumab as first‐line therapy. After initiation of second‐line therapy, median OS was not reached in the Cabo group and was 33 months (95% CI, 18 months–NA) in the Axi group (*p* = 0.415) (Figure 3a), while median PFS was 14 months (95% CI, 3–27 months) in the Carbo group and 12 months (95% CI, 7–29 months) in the Axi group (*p* = 0.504) (Figure 3b). There were no significant differences between the two groups.

### 3.3 Prognostic Factors

**FIGURE 4:**
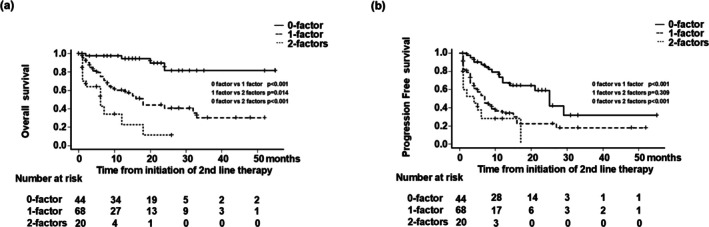
The group without the two prognostic factors C‐reactive protein ≥ 0.6 mg and estimated glomerular filtration rate < 40 mL/min/1.73 m^2^ was defined as 0‐factor group, with one of them as 1‐factor group and with both as 2‐factor group. (a) Kaplan–Meier curve analysis was used to compare overall survival (OS) after initiation of second‐line vascular endothelial growth factor receptor‐tyrosine kinase inhibitor treatment. Median OS was not reached, was 18 months, and was 6 months in the 0‐, 1‐, and 2‐factor groups, respectively, with significantly longer OS in the 0‐factor group (*p* < 0.001). (b) Progression‐free survival (PFS) after initiation of second‐line VEGFR‐TKI was also compared using Kaplan–Meier curve analysis. Median PFS was 25 months (95% CI, 12 months–NA), 7 months (95% CI, 4–10 months), and 4 months (95% CI, 1 month–NA) in the 0‐, 1‐, and 2‐factor groups, respectively, with significantly longer PFS in the 0‐factor group (*p* < 0.001).

We investigated the clinical parameters that predict prolonged OS after the initiation of second‐line VEGFR‐TKI therapy. Sex, Alb, serum CRP level, eGFR, and NLR were included as potential explanatory variables in the multivariate Cox proportional hazards model analysis. The Youden index obtained from the ROC curves was used to determine the cutoff values for serum Alb, CRP, eGFR, and NLR. The multivariable analysis identified CRP ≥ 0.6 mg (hazard ratio [HR], 4.23; 95% CI, 1.71–10.43; *p* = 0.001) and eGFR < 40 mL/min/1.73 m^2^ (HR, 2.59; 95% CI, 1.31–5.11; *p* = 0.005) as independent prognostic factors (Table 2). The group without these two prognostic factors was defined as the 0‐factor group, the group with either of these two prognostic factors was defined as the 1‐factor group, and the group with both of these prognostic factors was defined as the 2‐factor group. OS after initiation of second‐line VEGFR‐TKI was compared using Kaplan–Meier curve analysis. Median OS was NR (95% CI, NE–NE), 18 months (95% CI, 9–33 months), and 6 months (95% CI, 1–12 months) in the 0‐, 1‐, and 2‐factor groups, respectively, with significantly longer OS in the 0‐factor group (*p* < 0.001; Figure 4a). PFS after initiation of second‐line VEGFR‐TKI was also compared using Kaplan–Meier curve analysis. Median PFS was 25 months (95% CI, 12 months–NA), 7 months (95% CI, 4–10 months), and 4 months (95% CI, 1 month–NA) in the 0‐, 1‐, and 2‐factor groups, respectively, with significantly longer PFS in the 0‐factor group (*p* < 0.001; Figure 4b).

**TABLE 2:** Cox proportional hazards model of overall survival after initiation of second‐line therapy.

Variables	Multivariable		*p*
	HR	95% CI	
Gender (female vs. male)	1.01	0.471–2.156	0.982
Albumin (≥ 3.4 vs. 3.4 >)	0.55	0.276–1.102	0.092
CRP (≥ 0.6 vs. 0.6 >)	4.23	1.716–10.430	0.001
eGFR (< 40 vs. ≥ 40)	2.59	1.319–5.118	0.005
NLR (≥ 2.93 vs. 2.93 >)	1.56	0.688–3.558	0.284

Abbreviations: CI, confidence interval; CRP, c‐reactive protein; eGFR, estimated glomerular filtration rate; HR, hazard ratio; NLR, neutrophil‐lymphocyte ratio.

## 4 Discussion

Treatment strategies for mRCC have changed significantly in recent years, and although ICI combination therapy is now widely used as first‐line therapy, no evidence‐based regimen for sequential therapy, including second‐line therapy, has been proposed in several guidelines [14, 15]. First‐line therapy for mRCC includes nivolumab plus ipilimumab, a combination of two ICIs, and ICI‐TKI, an ICI plus a VEGFR‐TKI. With regard to second‐line therapy after ipilimumab plus nivolumab, Tomita et al. reported that the objective response rate (ORR) for the 19 Japanese patients enrolled in the Checkmate214 trial was 32%, and the median PFS after initiation of second‐line therapy was 16.3 months (95% CI, 11.0 months‐NR) [8]. Kojima et al. reported that axitinib was used in 77.3% of 22 patients who received a VEGFR‐TKI as second‐line therapy after nivolumab plus ipilimumab, with an ORR of 41.5% (95% CI, 26.3%–57.9%) and a median PFS after initiation of second‐line therapy of 17.8 months (95% CI, 5.6–25.8 months) after initiation of second‐line therapy [9]. In contrast, Iacovelli et al. evaluated second‐line therapy after ICI combination therapy and reported ORRs of 10%–80%, median OS of 11.8–24.9 months, and median PFS of 3.7–20.8 months for patients treated with ICI‐TKI as first‐line therapy [10].

In a retrospective report of outcomes in patients who received sunitinib as second‐line therapy after ICI combination therapy, the ORR of sunitinib with nivolumab plus ipilimumab and ICI‐TKI as first‐line therapy was 27.5% (95% CI, 13.0%–42.0%) and 20.8% (95% CI, 3.3%–38.4%), respectively (*p* = 0.55) [11]. The median OS after initiation of secondary treatment was 16.1 months (95% CI, 8.5%–35.2%) and 11.8 months (95% CI, 9.5%–NR), respectively (*p* = 0.65), with no significant difference between the two groups in either analysis [11]. In a comparative study of 48 patients who received cabozantinib as second‐line therapy after nivolumab plus ipilimumab and 60 patients who received axitinib, the ORRs were 37.5% and 38.3%, respectively [12]. Median OS was NR in the Cabo group (95% CI, 15.0 months–NE) and NR in the axitinib group (95% CI, 18.0 months–NE) (*p* = 0.932), while median PFS was 11.0 months (95% CI, 9.2–16.0 months) and 9.5 months (95% CI, 6.3–13.0 months) (*p* = 0.449), respectively, with no significant difference in oncologic outcomes between the two groups [12]. In a retrospective cohort study of patients with mRCC who received first‐line ICI combination therapy followed by second‐line VEGFR‐TKI therapy, the ORR for patients treated with nivolumab plus ipilimumab as first‐line therapy versus those treated with ICI‐TKI was 34.4% versus 25.9% (*p* = 0.26), median OS was 23.1 months versus 33.5 months (*p* = 0.93), median PFS was 9.7 months versus 7.1 months (*p* = 0.79), and there were no significant differences in oncologic outcomes between the two groups [13]. Although this study compared the oncological outcomes of second‐line treatment with VEGFR‐TKI after ICI combination therapy in the Cabo and Axi groups, there were no significant differences between the two groups. Furthermore, even when the first‐line treatment was limited to nivolumab plus ipilimumab, there was no significant difference in the median OS and PFS after the initiation of second‐line treatment between the Cabo and Axi groups. Thus, these results suggest that the efficacy of second‐line treatment with Cabo and Axi is equivalent in routine practice, regardless of the primary treatment regimen. Oncological outcomes were also likely to be similar when Cabo or Axi was used as an ICI combination therapy, regardless of the use of another agent. The use of ICI combination therapy overcomes the use of VEGF‐TKIs as first‐line therapy, thus improving the prognosis of mRCC patients compared to the era when only VEGFR‐TKIs were available. Furthermore, in this study, 44 out of 90 patients (52.4%) who received treatment with cabozantinib as second‐line therapy and 23 out of 45 patients (52.3%) who received treatment with axitinib as second‐line therapy received third‐line therapy after disease progression. Three types of TKIs, one type of mammalian target of rapamycin inhibitor (mTORi), and one type of ICI were used as treatment after second‐line cabozantinib therapy, and four types of TKIs and one type of ICI were used as treatment after second‐line axitinib therapy. In Japan, as of 2024, six types of VEGFR‐TKIs and two types of mTORi are available. Although there is insufficient evidence, the prognosis may be improved by using these molecular‐targeted drugs as sequential therapy in real‐world practice.

Regarding prognostic factors, Matsushita et al. reported CRP, Alb, and non‐clear cell histology as prognostic predictors for second‐line treatment with VEGFR‐TKI after first‐line treatment with ICI combination [13]. In our cohort, we performed a multivariable analysis of sex, CRP, Alb, NLR, and eGFR, which have been associated with prognosis in mRCC [22]. The results showed that eGFR and CRP were significant prognostic factors in this study. CRP has been an important prognostic factor in the history of drug therapy for mRCC [23, 24, 25]. Even in recent years, when immuno‐oncology drugs have become available, Yanagisawa et al. showed in a meta‐analysis of the prognosis of patients with metastatic renal cell carcinoma who received ICI therapy that high CRP levels are a biomarker of poor prognosis [25]. The evidence is accumulating that CRP is a prognostic factor after ICI combination therapy [13]. Previous studies of eGFR have used a cutoff of 60 mL/min/1.73 m^2^, suggesting that VEGFR–TKIs were used without affecting renal function [26, 27]. In this study, the cut‐off for eGFR was set at 40 mL/min/1.73 m^2^, demonstrating that impaired renal function is a potential predictor of worse oncologic outcomes, in contrast to the results of previously reported studies [26, 27]. As to why eGFR correlates with oncological outcomes, it has been suggested that cardiovascular disease complications and VEGFR‐TKI‐specific adverse events due to hypertension, diarrhea, and anorexia may lead to further decline in renal function, resulting in inadequate response to VEGFR‐TKI treatment [28, 29, 30].

Several limitations of this study have been identified. First, the study was retrospective and included a relatively small number of patients, which may have led to a potential selection bias. Second, the analysis was limited to Cabo and Axi as second‐line VEGFR TKIs. Therefore, no comparisons with other agents were performed. Hence, more effective sequential therapies are required. Third, the eGFR formula includes a correction for Japanese patients, which may lead to different results for other ethnic groups. Additionally, there may be a bias in the results because the study was conducted at values lower than 60 mL/min/1.73 m^2^, which is commonly used as a surrogate marker. Finally, this study did not collect data on adverse events associated with second‐line treatment with VEGFR TKIs. Therefore, the safety profile of the second‐line therapy could not be analyzed. This may be a challenge for future studies.

In conclusion, a multicenter retrospective study was conducted in 135 Japanese patients treated with Cabo or Axi therapy after ICI combination therapy. No significant differences in oncological outcomes such as OS and PFS after initiation of second‐line treatment were found between the two groups, suggesting that eGFR < 40 mL/min/1.73 m^2^ and CRP ≥ 0.6 mg/dL may be useful as predictors of prolonged OS after second‐line treatment with VEGFR‐TKI.

## Author Contributions

**Keita Nakane:** conceptualization, methodology, formal analysis, writing – original draft. **Hiromitsu Watanabe:** data curation. **Taku Naiki:** data curation. **Kiyoshi Takahara:** data curation. **Teruo Inamoto:** supervision, writing – review and editing. **Takahiro Yasui:** supervision, writing – review and editing. **Ryoichi Shiroki:** supervision, writing – review and editing. **Hideaki Miyake:** conceptualization, writing – review and editing. **Takuya Koie:** supervision, writing – review and editing.

## Ethics Statement

This study was approved by the Medical Review Board of Gifu University Graduate School of Medicine (approval number: 2024‐043).

## Consent

Due to the study's retrospective nature, the requirement for informed consent was waived; however, participants were given the option to opt out via the websites of all participating institutions. The need to obtain informed consent was waived because of the retrospective design; however, the ability to opt out was provided through the websites of all participating institutions.

## Conflicts of Interest

Hideaki Miyake received honoraria from Takeda, MSD, and Eisai. Teruo Inamoto received honoraria from Takeda, MSD, Pfizer, Ono, and Merck. Takuya Koie received honoraria from MSD. The other authors declare no conflicts of interest. Takahiro Yasui, Ryoichi Shiroki, and Hideaki Miyake are the Editorial Board members of the International Journal of Urology and the coauthors of this article. To minimize bias, they were excluded from all editorial decision‐making related to the acceptance of this article for publication.

## Supporting information


**Table S1.** Patient background who received nivolumab plus ipilimumab as first‐line therapy.

## Data Availability

The datasets in this study can be accessed by contacting the corresponding author upon reasonable request.
