# Supplementary material for: Impact of Estimated Glomerular Filtration Rate and Serum C‐Reactive Protein Level to Overall Survival After Second‐Line Targeted Therapy Following Immuno‐Oncology Combination Therapy for Advanced Renal Cell Carcinoma
Source: Int J Urol. 2025 May 28;32(9):1225–33. doi: 10.1111/iju.70138 (PMC12410124; doi:10.1111/iju.70138)
Supplement: Supplementary file 1 — Table S1. Patient background who received nivolumab plus ipilimumab as first‐line therapy. [file IJU-32-1225-s001.docx]

| **Supplementary Table 1** Patient background who received nivolumab plus ipilimumab as first-line therapy | | | |
| --- | --- | --- | --- |
| Patient background |  |  |  |
| **Characteristics** | **Cabozantinib** | **Axitinib** | ***P*** |
| number | 35 | 33 |  |
| Age at initiation of second-line therapy (median, year, IQR) | 67.0  (60.0 - 73.0) | 71.0  (62.0 - 75.0) | 0.236 |
| Sex (number, %) |  |  | 0.568 |
| Male | 27 (75.0) | 27 (81.8) |  |
| Female | 9 (25.0) | 6 (18.2) |  |
| BMI at initiation of first-line therapy (median, kg/m^2^, IQR) | 22.5  (21.0 – 24.9) | 22.3  (20.8 – 24.4) | 0.628 |
| KPS at initiation of first-line therapy (number, %) |  |  | 0.504 |
| 100 | 17 (47.2) | 12 (36.4) |  |
| 90 | 8 (22.2) | 9 (27.3) |  |
| 80 | 3 (12.6) | 7 (21.2) |  |
| 70 | 3 (10.3) | 3 (9.1) |  |
| Unknown | 0 | 1 (3.0) |  |
| Predominant histology (number, %) |  |  | 1.000 |
| Clear cell | 30 (83.3) | 30 (90.9) |  |
| Papillary | 1 (2.8) | 0 (0.0) |  |
| Chromophobe | 1 (2.8) | 1 (2.5) |  |
| Other | 4 (11.1) | 3 (9.1) |  |
| IMDC risk at initiation of first-line therapy (number, %) |  |  |  |
| Intermediate | 19 (52.8) | 18 (54.5) | 1.000 |
| Poor | 17 (47.2) | 15 (45.5) |  |
| Metastatic sites at initiation of first-line therapy (number, %) |  |  |  |
| Lung | 24 (66.7) | 26 (78.8) | 0.293 |
| Bone | 7 (19.4) | 10 (30.3) | 0.403 |
| Liver | 7 (19.4) | 4 (12.1) | 0.518 |
| Lymph node | 10 (30.3) | 8 (22.2) | 0.584 |
| Soft tissue | 0 (0.0) | 1 (2.8) | 1.000 |
| Adrenal gland | 4 (12.1) | 5 (13.9) | 1.000 |
| Brain | 2 (5.6) | 0 (0.0) | 0.494 |
| Pleura | 2 (6.1) | 0 (0.0) | 0.225 |
| Skeletal muscle | 0 (0.0) | 1 (2.8) | 1.000 |
| Local recurrence | 0 (0.0) | 1 (2.8) | 1.000 |
| Pancreas | 1 (3.0) | 1 (2.8) | 1.000 |
| C-reactive protein (median, mg/dL, IQR) | 0.6 (0.1-4.4) | 0.8 (0.3-2.8) | 0.657 |
| Serum Albumin (median, g/dL, IQR) | 3.9 (3.2-4.1) | 3.8 (3.4-4.0) | 0.939 |
| Serum Calcium (median, mg/dL, IQR) | 9.3 (9.0-10.1) | 9.6 (9.4-10.5) | 0.065 |
| eGFR (median, ml/min/1.73 m², IQR) | 51.3  (36.6-79.5) | 51.7  (37.2-66.8) | 0.960 |
| Previous surgical removal of primary site (number, %) |  |  |  |
| Yes | 23 (63.9) | 19 (57.6) | 0.629 |
| No | 13 (36.1) | 14 (42.4) |  |
| Follow up period (months, IQR) | 19.0  (11.2 - 31.0) | 28.0  (17.0 - 43.0) | 0.026 |
| IQR, interquartile range; BMI, body mass index; KPS, Karnofsky performance status; IMDC, international metastatic RCC database consortium; eGFR, estimated glomerular filtration rate | | | |
